# Mechanisms of Physical Activity Limitation in Chronic Lung Diseases

**DOI:** 10.1155/2012/634761

**Published:** 2012-12-12

**Authors:** Ioannis Vogiatzis, George Zakynthinos, Vasileios Andrianopoulos

**Affiliations:** ^1^Department of Physical Education and Sport Sciences, National and Kapodistrian University of Athens, 17237 Athens, Greece; ^2^Institute of Clinical Exercise and Health Science, University of West of Scotland, Hamilton ML3 0JB, UK; ^3^1st Department of Critical Care Medicine, National and Kapodistrian University of Athens, 10675 Athens, Greece; ^4^Thorax Foundation, Research Centre of Intensive and Emergency Thoracic Medicine, 10675 Athens, Greece

## Abstract

In chronic lung diseases physical activity limitation is multifactorial involving respiratory, hemodynamic, and peripheral muscle abnormalities. The mechanisms of limitation discussed in this paper relate to (i) the imbalance between ventilatory capacity and demand, (ii) the imbalance between energy demand and supply to working respiratory and peripheral muscles, and (iii) the factors that induce peripheral muscle dysfunction. In practice, intolerable exertional symptoms (i.e., dyspnea) and/or leg discomfort are the main symptoms that limit physical performance in patients with chronic lung diseases. Furthermore, the reduced capacity for physical work and the adoption of a sedentary lifestyle, in an attempt to avoid breathlessness upon physical exertion, cause profound muscle deconditioning which in turn leads to disability and loss of functional independence. Accordingly, physical inactivity is an important component of worsening the patients' quality of life and contributes importantly to poor prognosis. Identifying the factors which prevent a patient with lung disease to easily carry out activities of daily living provides a unique as well as important perspective for the choice of the appropriate therapeutic strategy.

## 1. Exercise Intolerance 

**Figure 1:**
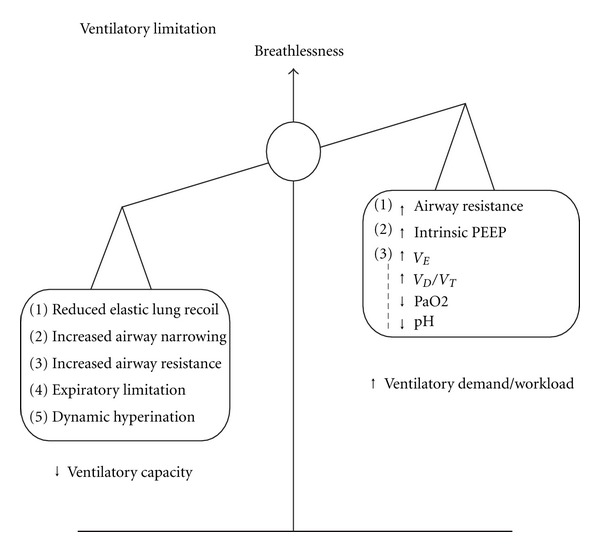
Conceptual framework of factors limiting exercise tolerance in COPD. *Mismatch of ventilatory capacity and ventilatory demand/workload*. Ventilatory capacity is reduced in patients with COPD and is thus insufficient to match the ventilatory requirement and increased workload. Such a mismatch leads to intense dyspnea sensations. (PEEP = positive end-expiratory pressure, *V*_*D*_/*V*_*T*_ = dead space/tidal volume).

Exercise intolerance is a condition where the individual is unable to perform physical exercise at the intensity or for the duration that would be expected of someone in his or her age and general physical condition. When this inability is caused by impaired function of one or more of the major physiological systems, namely the respiratory, the cardiovascular, and the peripheral muscle metabolic system, the result is the amplification of the perceptions of respiratory discomfort, either alone or typically in conjunction with peripheral muscle discomfort/fatigue [1]. In patients with chronic lung diseases, dyspnea sensations are exaggerated during exercise secondary to the reduced breathing efficiency that results from the deteriorating ventilatory mechanics on one hand and the increased ventilatory requirement on the other hand (Figure 1).

Respiratory discomfort is typically perceived as the distressing sensation of unsatisfied inspiration because of a mismatch between central neural drive and the respiratory mechanical/muscular response (i.e., the so-called “neuromechanical uncoupling or dissociation”) of the respiratory system [2]. Patients with chronic lung diseases constantly select descriptor clusters that allude to both “increased respiratory work/effort” and “unsatisfied inspiration” upon cessation of physical exercise. Recent theories on the mechanisms of respiratory discomfort have emphasized the central importance of the perception of increased contractile inspiratory muscle effort (dyspnea perceived as “increased respiratory effort”) [3]. In fact, inspired effort and central motor command output are both increased compared to healthy individuals, reflecting the relatively higher ventilation, as well as the increased loading and functional weakness of the inspiratory muscles [4, 5]. Particularly in patients with chronic obstructive pulmonary disease (COPD) altered afferent information from activated mechanoreceptors in the overworked and shortened inspiratory muscles, secondary to dynamic lung hyperinflation, may contribute to an increased sense of work or effort, but this remains speculative [6, 7]. It has long been suggested that in these patients, a mismatch between central neural drive and the respiratory mechanical/muscular response (“neuromechanical uncoupling” or dissociation) of the respiratory system, as crudely reflected by the increased effort-displacement ratio, is fundamental to the origin of perceptions of unrewarded inspiratory effort (i.e., “unsatisfied inspiration”) [2, 8]. 

Peripheral muscle contractile fatigue occurring secondary to a limitation in oxygen supply to, and/or utilization of oxygen by, the mitochondria [9] also constitutes an important factor that limits exercise capacity in patients with chronic lung diseases. This suggestion is further supported by the finding that the degree of exercise-induced quadriceps muscle fatigue in COPD negatively correlates with peak oxygen utilization [10]. A decrease in locomotor muscle force output compared to the predicted normal values has also been reported in patients with interstitial lung disease (ILD) [11] and pulmonary arterial hypertension (PAH) [12].

Accordingly, it is likely that cellular oxygen demand either exceeds the normal maximal oxygen transfer capacity of the oxygen transport chain, (i.e., when maximal oxygen consumption has been truly achieved), or stresses an impaired physiological system (i.e., cardiovascular and/or respiratory) preventing the achievement of a true maximal oxygen consumption. Hence, the factors that limit physical performance in healthy individuals (i.e., when oxygen demand exceeds the normal maximal oxygen transfer capacity) are different to those (i.e., impairment in oxygen transport) constraining the capacity to perform physical exercise in patients with chronic lung diseases (i.e., ventilatory limitation) [9, 13]. 

## 2. Physiological Factors Impairing Physical Activity 

Exercise intolerance in patients with chronic lung diseases is multifactorial, involving ventilatory, gas exchange, cardiovascular, and peripheral muscle abnormalities. 

### 2.1. Ventilatory Constraints

During incremental exercise, healthy elderly individuals can sufficiently increase their breathing frequency and their tidal volume to provide up to a 10–15 fold increase in minute ventilation that is essential to clear the carbon dioxide production and meet the increased oxygen demand [14–17]. Under such circumstances, ventilatory function is often not the limiting factor, at least for a wide range of submaximal exercise levels, as minute ventilation (${\dot{V}}_{E}$) is maintained well below the maximum ventilatory capacity (MVC) [18]. Ventilatory limitation, however, may occur in healthy elderly individuals, particularly women [19] during maximal exercise, as the ratio of ${\dot{V}}_{E}$ to MVC (${\dot{V}}_{E}/\text{MVC}$) approaches or even exceeds 85% [20, 21]. While an increased ratio (i.e., >85%) of peak exercise ventilation to the estimated MVC strongly suggests limiting ventilatory constraints, a preserved peak ${\dot{V}}_{E}$/MVC ratio (i.e., <75% predicted) by no means excludes the possibility of significant ventilatory impairment during intense physical activity [22–26].

Patients with chronic lung diseases are deemed to have ventilatory limitation if, at cessation of exercise, the patient has reached estimated MVC, while at the same time cardiac and other physiological functions are operating below maximal capacity. Hence, attendant respiratory discomfort may limit exercise capacity before actual physiological limitation occurs, and the relative importance of other nonventilatory factors is impossible to quantify with precision. Thus, simultaneous analysis of exercise flow-volume loops at the point of exercise limitation may show marked constraints on flow and volume generation in the presence of an apparently adequate ventilatory reserve as estimated from the peak ${\dot{V}}_{E}$/MVC ratios [22–26]. In a recent study, 14% of a population sample of clinically stable patients with COPD (*n* = 105), with apparent ventilatory reserve at peak exercise (i.e., ${\dot{V}}_{E}/\text{MVC}<75\%$  predicted) had coexisting limiting restrictive ventilatory constraints [i.e., behaved as if they had a restrictive ventilatory defect due to constrained ability to increase *V*_*T*_ when end-inspiratory lung volume (EILV) approached total lung capacity (TLC)] as indicated by an EILV >95% of TLC that is, significantly reduced peak inspiratory reserve volume (IRV) at the same time point [24]. In addition, significant ventilatory constraints may be detected on exercise flow-volume loop analysis, even in patients with mild COPD [22, 25, 26] who have an apparently normal ventilatory reserve at peak exercise, as ascertained again by the peak ${\dot{V}}_{E}$/MVC method. Therefore, the role of exercise flow-volume loop analysis combined with the behavior of dynamically assessed operating lung volumes is crucial in ascertaining the presence or not of significant ventilator constraints in all chronic pulmonary diseases.

In the majority of patients with chronic lung diseases, a disparity is developed between the decreased ventilatory capacity, which is manifested by diminished maximum and sustainable voluntary capacity and eventually by the inability to sufficiently increase minute ventilation during intense physical activities, and the increased ventilatory requirement of exercise [14, 27]. This disparity leads to intense dyspnea sensation that is the symptom limiting physical activity in a large fraction of patients with chronic lung diseases [28, 29]. The factors contributing to decreased ventilatory capacity or increased ventilatory requirement/workload are in brief described below (Figure 1). 

#### 2.1.1. Reduced Ventilatory Capacity

Reduced ventilatory capacity during intense physical activity is due to the abnormal respiratory system mechanics and the dysfunction of the respiratory muscles. In patients with chronic lung diseases, the high inspiratory (and expiratory) airway resistance and/or reduced lung compliance (that occurs in ILD and in COPD when breathing on the flat portion of the pressure/volume relationship) can substantially increase the pressure requirement for airflow and thus increase the work of breathing [29–31]. Respiratory muscles are frequently weakened and unable to endure a given workload adequately due to the presence of hyperinflation and/or intrinsic muscle dysfunction/hypoperfusion. 

#### 2.1.2. Ventilatory Demand

**Figure 2:**
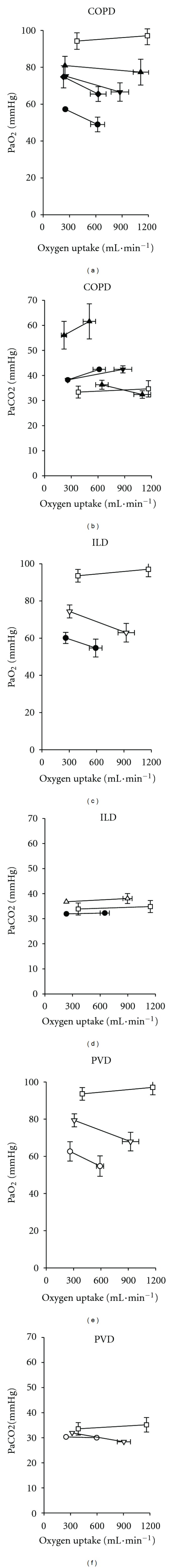
Mean arterial oxygen and carbon dioxide tension during exercise in chronic lung diseases. Arterial oxygen tension (PaO_2_) and carbon dioxide tension (PaCO_2_) as a function of oxygen uptake at rest and during high-intensity exercise in COPD (a and b; range of *n* = 7 to 23); ILD (c and d; range of *n* = 8 to 12); PVD (e and f; rage of *n* = 7 to 11). Exercise usually causes PaO_2_  to fall in all three diseases. PaCO_2_ often rises in COPD but falls or does not change in ILD, PVD, and healthy subjects (□). *With permission from Agusti et al., 1997 *[43].

**Figure 3:**
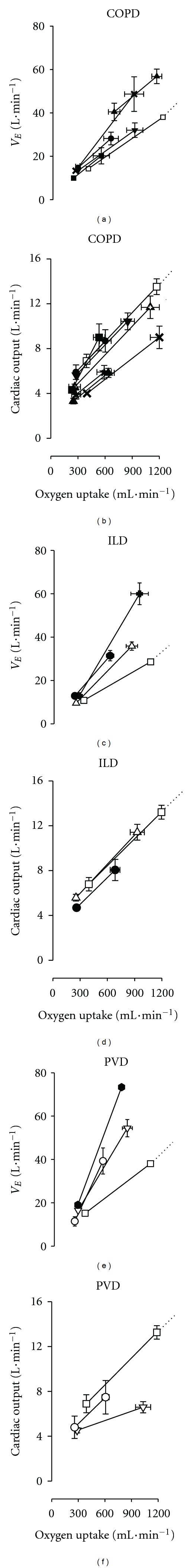
Mean minute ventilation and cardiac output during exercise in chronic lung diseases. Minute ventilation (*V*_*E*_) and cardiac output as a function of oxygen uptake at rest and during exercise in patients with COPD (a and b), ILD (c and d), PVD (e and f), and healthy subjects (□). *Adapted with permission from Agusti et al., 1997 *[43].

Ventilatory demand is increased during intense physical activity owing to gas exchange abnormalities (i.e., worsening of alveolar ventilation/perfusion [${\dot{V}}_{A}/Q$] mismatch and increased dead space ventilation) which lead to hypoxemia and hypercapnia [20]. The ventilatory demand of exercise is regulated not only by the metabolic rate but also by the arterial carbon dioxide tension (PaCO_2_) and the physiological dead space fraction of breath [32]. Metabolic acidosis also increases the ventilatory requirement of intense physical activity [33]. Therefore, in chronic lung diseases, for a given rate of CO_2_ output (${\dot{V}}_{\text{C}{\text{O}}_{2}}$) and PaCO_2_, ${\dot{V}}_{E}$ is usually increased because of higher dead space ventilation [1] (Figures 2 and 3). Moreover, *ventilatory workload is increased *during exercise because of abnormal dynamic ventilatory mechanics.

**Figure 4:**
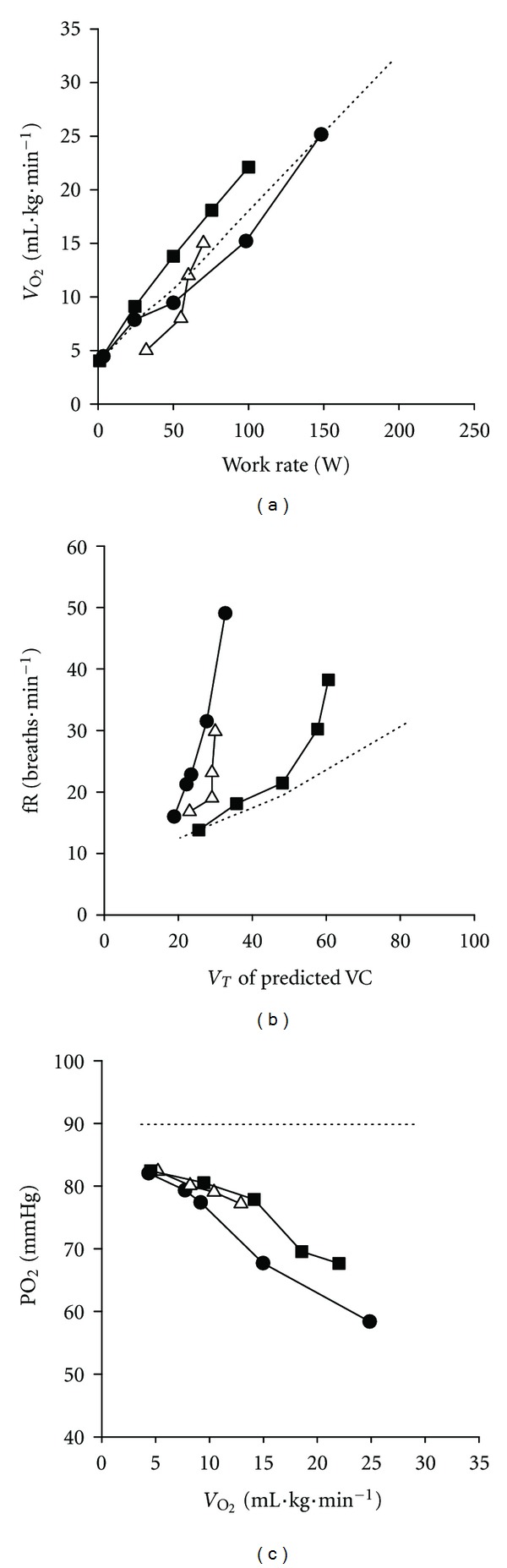
Ventilatory and gas exchange responses during incremental exercise in chronic lung diseases. Typical exercise responses in COPD (▵) interstitial lung disease (ILD: ●), PVD (■), and healthy age-matched subjects (- - - - - -) for (a) oxygen uptake (b) respiratory frequency (fR), and (c) arterial oxygen tension. Responses plotted as a function of oxygen uptake (${\dot{V}}_{{\text{O}}_{2}}$), work rate, or tidal volume as a % of predicted vital capacity (VC). *With permission from O'Donnell et al., 2007 *[3–5].

In practical terms, during incremental cardiopulmonary exercise testing, exercise intolerance in patients with COPD is typically manifested by reduced peak oxygen uptake (Figure 4(a)) and an early occurrence of the lactate threshold secondary to premature lactic acidosis [14, 33–38]. Early termination of exercise is also accompanied by low peak ${\dot{V}}_{E}$, substantial ventilatory inefficiency (marked by increased ventilatory equivalents for carbon dioxide), and decreased ventilatory reserve (i.e., evident by increased peak  ${\dot{V}}_{E}/\text{MVC}$). 

Typically, minute ventilation increases progressively with increasing exercise intensity in COPD in such a manner that the relationship between ventilation and work rate or oxygen uptake often has a sharper slope when compared to that recorded in healthy age-matched individuals. This is because at a given level of external submaximal intensity, minute ventilation is higher than in healthy subjects owing to increased dead space ventilation (Figure 3(a)). Consequently, at a given work or metabolic rate COPD patients endure a considerably greater work of breathing than their healthy counterparts owing to the higher ventilatory rate *per se* and also to the higher cost per liter of ventilation. The latter is due to the fact that abnormal dynamic ventilatory mechanics of COPD require a greater degree of effort to move a given volume of air. 

Expansion of tidal volume is also restricted secondary to the development of dynamic hyperinflation, whereas breathing frequency is increased (Figure 4(b)). Repeated measurements of inspiratory capacity during exercise demonstrate a progressive decrease in this variable indicating that end-expiratory lung volume has been increased [39, 40]. 

In summary, the deteriorating ventilatory mechanics and the increased ventilatory requirement occurring even during mild to moderate physical exertion in patients with chronic lung diseases worsens breathing efficiency, thereby exaggerating dyspnea sensations.

### 2.2. Gas Exchange Limitations

Age-related changes in pulmonary circulation would be expected to make elderly individuals more susceptible to gas exchange abnormalities during exercise. However, despite the deterioration in ventilatory reserve with aging, healthy older adults appear able to maintain alveolar ventilation at a level that allows maintenance of arterial blood gases within normal limits, even during heavy exercise [5, 18, 20, 41]. Accordingly, ${\dot{V}}_{A}/Q$  remains near unity as both ventilation and perfusion increase several-fold with increasing intensity of physical activity. Moreover, alveolar-capillary diffusion also remains intact, and consequently PaO_2_ remains normal, even at a high-intensity physical activity [14, 18, 20] (Figure 2(a)). Furthermore, in healthy elderly individuals, exercise-induced tidal volume (*V*_*T*_) increase occurs in the setting of relatively fixed anatomic dead space (*V*_*D*_), so the *V*_*D*_/*V*_*T*_ ratio decreases such that effective alveolar ventilation increases as a proportion of the increased minute ventilation. 

In contrast, gas exchange regulation is impaired in chronic lung diseases that involve the airways, the pulmonary vasculature, and the alveolar-capillary interface to varying degrees thereby producing varying degrees of abnormal  ${\dot{V}}_{A}/Q$  inequalities, diffusion impairment, and hypoxemia during exercise. In fact, many patients with severe lung disease experience arterial oxygen desaturation during exercise. Furthermore, in Chronic Lung Diseases that affect the pulmonary vasculature, arterial PCO_2_ may be higher than in healthy subjects as *V*_*D*_ is increased owing to reduced  ${\dot{V}}_{A}$  [3, 29, 42, 43] (Figure 2(b)).

Measurement of physiological dead space (*V*_*D*_ physiological) requires the assumption that the PCO_2_ of the exchanging (i.e., perfused) alveoli equals PaCO_2_. Normally *V*_*D*_ physiological is approximately equal to anatomical dead space (*V*_*D*_ anatomical) and accounts for about 25%–30% of *V*_*T*_ at rest. It is increased with exercise, consequent to the expanding influence on the conducting airways of the greater transpulmonary pressures. However, as the expansion of the alveolar space is appreciably greater than that of the less distensible conducting airways, *V*_*D*_ physiological/*V*_*T*_ falls (typically to ~0.1-0.2 at peak exercise). Naturally, *V*_*D*_ physiological/*V*_*T*_ is appreciably larger than *V*_*D*_ anatomical in many pulmonary diseases, with *V*_*D*_ physiological/*V*_*T*_ being as high as 0.5. 

Due to the early termination of exercise, peak heart rate is relatively low whilst the heart rate reserve is high. In addition, patients often exhibit arterial hypoxemia (Figure 4(c)) that is manifested by a decrease in arterial oxygen saturation. Furthermore, owing to the reduced alveolar ventilation during exercise, hypercapnia emerges reflecting overt ventilatory insufficiency. In addition, in patients with COPD mechanical factors may substantially constrain the ventilatory response to the metabolically generated CO_2_ in such a way that PaCO_2_ may not decrease as in normal healthy subjects (i.e., compromised respiratory compensation) [44–47] (Figure 2(b)). Indeed, severe mechanical restriction secondary to dynamic hyperinflation and increased respiratory muscle work in a setting of an increased physiological dead space has been recognised as a contributory factor to hypercapnia in COPD [48]. 

In summary, during prolonged exercise the aforementioned gas exchange abnormalities worsen the alveolar ventilation/perfusion inequalities further increasing dead space ventilation that in turn leads to hypoxemia and hypercapnia. All these factors contribute to increased ventilatory requirement that in the face of reduced ventilator capacity exaggerate dyspnea sensations, thereby compromising physical capacity.

### 2.3. Central and Peripheral Hemodynamic Factors

Cardiac output in healthy elderly subjects can increase several-fold in response to exercise [5, 18, 20, 49, 50]. In the majority of healthy elderly subjects, cardiac output is often the “rate-limiting step” to exercise, and normal maximal exercise is usually accompanied by a heart rate that often approaches the maximal predicted. In contrast, in chronic lung diseases, the following mechanisms that involve oxygen transport are frequently impaired resulting in reduction of cardiovascular function (Figure 3). Firstly, coexisting right or left ventricular dysfunction can impair physical activity simply because of poor cardiac output capability, which often leads to impaired oxygen delivery and early development of metabolic acidosis. Similarly, functionally important arrhythmias may also impair the normal increase in cardiac output as a function of an increase in work rate [5, 18, 20, 51]. Secondly, in chronic lung diseases, especially in the presence of pulmonary vascular abnormalities, pulmonary hypertension and right ventricular dysfunction may develop [52]. The impaired right ventricle may thus contribute to a limited increase in cardiac output. These phenomena may worsen in the presence of hypoxemia. Hypoxemia can in turn elevate pulmonary vascular resistance and create pulmonary arterial hypertension with consequent right heart failure [43, 52–58]. The resulting restrained increase in cardiac output, coupled with the low oxygen content, reduces systemic oxygen delivery to all organs of the body, including skeletal muscles. Interestingly, because the work of breathing is often substantially increased in chronic lung diseases, there might also exist a respiratory muscle “steal” of blood flow away from the locomotor muscles, which further compromises peripheral muscle function [59, 60]. 

In COPD, constant-load exercise tolerance has been documented to largely depend on the imposed workload as the time to the limit of tolerance decreases (similarly to normal subjects) hyperbolically as a function of power output [61]. The hyperbolic shape of the power output-endurance time relationship has been shown to be determined by the dynamics of the ventilatory response toward a reduced and fixed maximum ventilatory ceiling. More explicitly, for each individual COPD patient there is a so-called “critical power” that represents the highest work rate at which there is sufficient ventilatory reserve. In terms of physiological responses, studies [62, 63] have revealed that in the transition from rest to constant-load exercise, pulmonary oxygen uptake kinetic responses are slower in patients with COPD as compared to age-matched healthy individuals. This sluggishness of oxygen uptake is thought to lead to an early and greater reliance on oxygen-independent metabolic pathways and accumulation of by-products that accelerate the occurrence of muscle fatigability. Along these lines, there is uniform agreement [14, 33–38] that lactic acid production occurs at a very low level of physical activity in COPD (i.e., <40% predicted peak ${\dot{V}}_{{\text{O}}_{2}}$) [27, 60, 64]. In addition, derangements in the diffusive and convective transport of oxygen to skeletal muscle mitochondria have been portrayed as plausible factors to delayed pulmonary oxygen uptake kinetics [62, 63, 65]. Chiappa and colleagues [66] extended those findings by showing that COPD patients also display slower cardiac output kinetics along with faster dynamics of vastus lateralis muscle deoxygenated haemoglobin (an index of muscle microvascular oxygen extraction). These findings have been interpreted to indicate impaired central and peripheral muscle hemodynamic adjustments in COPD compared to healthy subjects [66, 67]. 

In summary, in chronic lung diseases cardiovascular factors associated with coexisting right and/or left ventricular dysfunction, functional arrhythmias, and various negative cardiopulmonary interactions can impair cardiac function and thus physical activity. 

### 2.4. Skeletal Muscle Abnormalities

Regardless of overt respiratory insufficient, patients with chronic lug diseases are commonly characterized by reduced physical activity. Inactivity in COPD leads to muscle weakness and altered muscle fibre distribution [68] reflected by high proportion of type I slow twich fibres which are highly oxidative, low tensioned, and fatigue resistant [12, 69–74]. Reduction in the proportion of oxidative fibres reduces the oxidative potential of the muscles and would make them more vulnerable to fatigue during high-intensity exercise. There is also less capillary density that reduces regional blood flow and oxygen/nutrient delivery. Such structural and metabolic abnormalities of the limb muscles may lead to early lactic acidosis and task failure with exercise [69–73].

In addition, systemic inflammatory mediators, which can profoundly affect skeletal muscle function [44, 75–77] are persistently elevated in chronic lung diseases, thereby accelerating muscle protein degradation [78–85]. This contributes to the loss of muscle mass and the clinical appearance of “muscle wasting” [44, 75–77, 85–87]. Chronic inflammation also increases muscle oxidative stress and increases reactive oxygen species, which directly damage muscle proteins, impair their function, and lead to protein degradation [29, 88–90].

Furthermore, patients with chronic lung diseases [27] are often malnourished. Weight loss occurs in approximately 30% of out-patients with chronic lung diseases [91–96], because of decreased calorie intake and the effects of chronic inflammation on energy metabolism in general. Reduced protein intake leads to muscle breakdown as muscle proteins and amino acids are utilized for fuel (catabolism). Malnutrition also contributes to reduced muscle enzyme capacity and reduced availability of energy substrates [97–101]. Finally, patients with chronic lung disease may also take corticosteroids, particularly during exacerbations. Corticosteroids can profoundly affect skeletal muscle, as they reduce contractile proteins, increase protein breakdown and turnover, downregulate growth factors, reduce glycolytic activity, and lead to sarcomere and type-II fibre atrophy [102–104]. 

Accordingly, lower limb muscles in patients with chronic lung diseases are atrophied, weak, fatigable, and metabolically inefficient. These unfavorable muscle characteristics concur to limit exercise capacity, a most debilitating feature in these patients.

In summary, although several mechanisms underlying the development of skeletal muscle dysfunction have been identified (e.g., deconditioning), it is important to further identify the impact of other potential contributors to skeletal muscle dysfunction in chronic lung diseases (such as inflammation, malnutrition, oxidative stress, and inflammation, etc).

### 2.5. Exercise Intolerance in Chronic Lung Diseases

In patients with COPD, exercise intolerance involves respiratory mechanical, pulmonary gas exchange, hemodynamic and peripheral muscle abnormalities that interfere with the normal in-series system (ventilation, gas exchange, blood flow, hemoglobin, muscle O_2_/CO_2_ transport, and O_2_ utilization/CO_2_ production) upon which exercise depends, thereby ultimately preventing adequate oxygen transfer from the atmosphere to, and/or utilization of oxygen by, the mitochondria. Such abnormalities may occur consequently due to the following reasons: (i) limited ventilatory capacity to suffice the ventilatory requirement, (ii) imbalance between the high blood/oxygen requirement of the locomotor and/or respiratory muscles and the limited blood/oxygen supply to these muscles, and (iii) dysfunction/weakness and reduced oxygen utilization capacity at the level of the peripheral muscles [1–13].

The primary mechanisms limiting exercise tolerance in patients with ILD include the restrictive lung mechanics, pulmonary gas exchange derangements, hemodynamic abnormalities, and peripheral muscle dysfunction [4]. Ventilatory inefficiency occurs secondary to high physiological dead space and arterial hypoxemia and thirdly to premature metabolic acidosis. Likewise, the oxygen cost of breathing per unit ventilation is increased in patients with ILD as the static recoil pressure of the lungs is increased, thereby requiring greater inspiratory muscle activity [43]. Impaired gas exchange occurs as a result of destruction of the pulmonary capillary bed or thickening of the alveolar capillary membrane, causing ventilation/perfusion mismatch, oxygen diffusion limitation, and low mixed venous partial pressure of oxygen. Circulatory limitation resulting from pulmonary capillary destruction and hypoxic vasoconstriction leading to pulmonary hypertension and cardiac dysfunction also plays an important role in exercise limitation [43].

Relatively reduced lung compliance and inspiratory muscle weakness have been suggested as potential contributing factors of abnormal ventilatory mechanisms in pulmonary vascular diseases. During exercise, there is substantial arterial oxygen desaturation causing widening of alveolar-arterial oxygen tension, reflecting considerable  VA/*Q*  inequalities. Circulatory limitation resulting from pulmonary capillary destruction and hypoxic vasoconstriction leading to pulmonary hypertension and cardiac dysfunction also plays an important role in exercise limitation [3, 43].

## 3. Conclusions

The available literature suggests that in the majority of patients with severe chronic lung disease, primarily ventilatory constraints, resulting from the imbalance between ventilatory demand and capacity, limit physical capacity due to intense dyspnea sensations, whereas to a lesser extent, inadequate energy supply to locomotor muscles and/or locomotor muscle dysfunction limit(s) physical activity performance secondary to locomotor muscle discomfort. In contrast, in many patients with mild and moderate chronic lung disease, both reduced energy supply to locomotor muscles associated with leg discomfort and ventilatory constraints causing breathlessness restrain exercise tolerance.
